# Baicalin-modified polyethylenimine for miR-34a efficient and safe delivery

**DOI:** 10.3389/fbioe.2023.1290413

**Published:** 2023-11-03

**Authors:** Yingying Wang, Baiyan Wang, Yangfan Xiao, Qingchun Cai, Junyue Xing, Hao Tang, Ruiqin Li, Hongtao Zhang

**Affiliations:** ^1^ Medical College, Henan University of Chinese Medicine, Zhengzhou, Henan, China; ^2^ National Health Commission Key Laboratory of Cardiovascular Regenerative Medicine, Heart Center of Henan Provincial People’s Hospital, Fuwai Central China Cardiovascular Hospital and Central China Branch of National Center for Cardiovascular Diseases, Central China Fuwai Hospital of Zhengzhou University, Zhengzhou, Henan, China; ^3^ Department of Clinical Lab, The Third Affiliated Hospital of Henan University of Chinese Medicine, Zhengzhou, China; ^4^ Academy of Chinese Medicine, Henan University of Chinese Medicine, Zhengzhou, Henan, China; ^5^ Blood Purification Center, The People’s Hospital of Zhengzhou University, Zhengzhou, China; ^6^ Blood Purification Center, Henan Provincial People’s Hospital, Zhengzhou, China; ^7^ Institute of Nephrology, Mathura, Henan, China; ^8^ Department of Nephrology Henan Provincial People’s Hospital, Zhengzhou, China

**Keywords:** baicalin, lung cancer, gene therapy, miR-34a, hydrophobic modification

## Abstract

The security and efficiency of gene delivery vectors are inseparable for the successful construction of a gene delivery vector. This work provides a practical method to construct a charge-regulated, hydrophobic-modified, and functionally modified polyethylenimine (PEI) with effective gene delivery and perfect transfection performance through a condensation reaction, named BA-PEI. The carrier was shown to possess a favorable compaction of miRNAs into positively charged nanoparticles with a hydrodynamic size of approximately 100 nm. Additionally, BA-PEI possesses perfect degradability, which benefits the release of miR-34a from the complexes. In A549 cells, the expression level of the miR-34a gene was checked by Western blotting, which reflects the transfection efficiency of BA-PEI/miR-34a. When miR-34a is delivered to the cell, the perfect anti-tumor ability of the BA-PEI/miR-34a complex was systematically evaluated with the suppressor tumor gene miR-34a system *in vitro* and *in vivo*. BA-PEI-mediated miR-34a gene transfection is more secure and effective than the commercial transfection reagent, thus providing a novel approach for miR-34a-based gene therapy.

## 1 Introduction

In the world, cancer, which accounts for one in six deaths, is one of the deadliest diseases (WHO, 2023). Due to the characteristic of lung cancer to metastasize easily, the death rate of lung cancer is the leading cause of mortality among all kinds of malignant tumors (IARC, 2020). Vaccination, initial diagnosis, and appropriate treatment are important measures to reduce cancer mortality. However, due to the variability and complexity of the tumor, these methods have a low success rate. Surgery and radiation therapy are used to treat orthotopic tumors, while anti-tumor agents, such as chemotherapy, hormonal therapies, and biotherapies, are preferred for metastatic tumors. With the increasing advancement of molecular biology and the understanding of unique tumor characteristics, chemotherapy drugs can induce apoptosis of normal cells and are toxic, so gene therapy is a promising targeted therapy for cancer.

Gene therapy involves the introduction of foreign nucleic acids, such as genes, gene fragments, oligonucleotides, miRNAs, or siRNAs, into target cells and regulating the synthesis of target genes, mRNAs, or foreign proteins (Xin et al., 2017; Ferrari et al., 2021; Hu et al., 2021; Wang et al., 2021; Paunovska et al., 2022). The most studied cancers include those of the liver, pancreas, breast, lung, colorectal, and prostate (Chen et al., 2019; Haffner et al., 2021; Khan et al., 2021; Yu et al., 2021; Hong and Xu, 2022). miR-34a causes cell cycle arrest, promotes cell senescence, and induces cell apoptosis and performs a function that prevents cell migration. In the occurrence and progression of tumors, the reason for tumor resistance to chemotherapeutic agents, which exert anti-tumor effects through p53, is that miR-34a is deactivated by the methylation of CpG islands (Kim and Kim, 2014; Qian et al., 2014). Studies indicate that the ectopic augmentation of miR-34a inhibits glioma cell proliferation and promotes G1/S phase cell cycle arrest. The success of cancer gene therapy depends not only on sound molecular strategies, including the construction of a factual genetic material specifically expressed in tumor cells, but also on a safe, highly effective, and controlled vector preparation (Raguram et al., 2022).

Viral vectors were the first vectors proposed for gene therapy. The properties and characteristics of viruses make them ideal vectors for delivering RNA and DNA to human cells, and a number of clinical trials have been carried out, resulting in the approval of some gene therapy drugs (Lundstrom, 2018). However, immunogenicity, limited genetic loads, cancer due to the insertion of a therapeutic payload near a gene that controls cell growth, and the risk of viral vector mass production have promoted the development and engineering of non-viral vectors supported by nanomedicine and facilitated the development and engineering of nanomedicine-supported non-viral vectors (Kaiser, 2020). Nowadays, several cationic polymers are being developed, due to their facile synthesis, low host immunogenicity, and perfect transfection performance, such as polyethylenimine (PEI) for gene delivery (Kim and Kim, 2014; Yamano et al., 2014), methacrylate-based cationic copolymer (Li et al., 2015), and poly (L-lysine) (Wang, 2009). For non-viral gene transfer, a 25 kDa branched PEI is the golden standard. However, its long-term toxicity and low biocompatibility due to excessive positive charge have become the key limitations to its clinical application.

Baicalin (BA), which can efficaciously inhibit cancer cell proliferation and induce its apoptosis, is the main component of *Scutellaria baicalensis* (SB) (Shou et al., 2017; Duan et al., 2019; Han et al., 2021). It was found that baicalin could activate SIRT1/AMPK signaling in A549 and H1299 cells, inhibiting their proliferation and migration. Moreover, baicalin, inducing cell cycle arrest and promoting cell apoptosis, chiefly exerts its anti-tumor activity in an Akt-dependent manner. Baicalin, as an effective component of traditional Chinese medicine Scutellaria, can be used as an effective anti-cancer drug. However, its effective components are delivered to the cells for further study. In this work, a BA-decorated PEI was synthesized to achieve positive charge density equilibrium. Then, miR-34a was selected as the therapeutic gene and BA-PEI was used as a carrier. In this system, miR-34a also cooperates with baicalin to exert an anti-tumor effect and enhance the curative effect. Moreover, baicalin can effectively bond with PEI to form a new complex, BA-PEI. The miR-34a genetic drug was integrally appraised by the inhibition of tumor proliferation and migration using A549 and tumor-bearing mice as models.

## 2 Methods

### 2.1 Hemolysis assay with the blood sample

In brief, the blood sample was collected in EDTA tubes to avoid clotting, and RBCs were extracted by centrifuging the blood at 1,500 rpm for 10 min. We removed the supernatant and re-suspend the erythrocytes in PBS to a density of 5.0 × 109 cells/mL. The suspension (100 μL) was incubated with a nanoparticle solution (900 μL) at 37°C for 30 min and centrifuged at 1,500 rpm for 10 min to obtain the supernatant. The absorbance of hemoglobin release was measured using the Varioskan^TM^ LUX microplate reader (Thermo, United States) at 578 nm. Hemolysis was carried out based on the following formula:

$$Hemolysis\;\left(\%\right)=\frac{Sample\;OD578-NC\;OD578}{PC\;OD578-NC\;OD578}\times 100\%.$$

### 2.2 Cell apoptosis analysis

The cells were seeded and transfected as previously mentioned; then, Annexin V-FITC and PI were used to stain the treated cells, adhering to the instructions. The effect of apoptosis was evaluated by flow cytometry using a Canto II (BD, CA).

### 2.3 Cell colony experiment

Similarly, the cells were planted and transfected, as mentioned previously. 1 × 103 cells that had been cultured for approximately 7 days were redigested. The colonies were washed with 1 × PBS, fixed with cold 70% methanol, and stained with 0.5% crystal violet. Finally, we washed them twice with PBS and observed them using a fluorescence microscope, IX73 (OLYMPUS, Japan).

### 2.4 Wound healing assays

The A549 cells were seeded as described previously. Untreated cells were straightly scratched in the middle using a yellow pipette tip. Then, different nanoparticles were transferred and incubated in the corresponding wells. The FBS-free 1640 medium was utilized for 6 h and replaced by a complete 1640 medium. Meanwhile, the stationary view of the wound was measured by width and photographed via a microscope, IX73 (OLYMPUS, Japan).

### 2.5 Western blot assay

We planted and transfected the A549 cells, as previously mentioned. RIPA lysed the harvested cells to gather the total protein, and the protein concentration was measured using a BCA protein detection kit. The identical proteins were assayed using SDS-PAGE and transferred to the PVDF membrane in the running buffer and the transferring buffer, respectively. The membrane was treated with a 5% non-fat milk solution for 1 h to block background proteins at room temperature. The membrane was incubated with primary antibodies at 4°C overnight and HRP-conjugated secondary antibodies at room temperature for 1 h, successively. The membrane was observed using the Tanon 4800 (Shanghai, China) using an ECL reagent and analyzed using ImageJ.

### 2.6 Establishment of the tumor-bearing model

The subcutaneous xenograft tumor-bearing model was established by BALB/c nude mice, which were acquired from Vital River Laboratory Animal Technology Co., Ltd. (Beijing, China). Furthermore, A549 cells were subcutaneously injected into the right side of the mice at a concentration of 1.0 × 10^7^ cells/mouse. The tumor volume was measured as length × (width) 2/2 and recorded along with the mice’s body weight during the period. When the tumor volumes reached 50 mm^3^, a different nanoparticle was injected into the tail vein every 3 days at a concentration of 0.5 mg/kg body weight.

### 2.7 Statistical analysis

All data were expressed as mean ± standard deviation, and unidirectional analysis of variance with GraphPad Prism 6 and *t*-tests were used to statistically test the differences between different experimental groups and control groups (ns, no significant; *p <0.05; **p <0.01).

## 3 Results and discussion

### 3.1 Synthesis and characterization of BA-PEI/miR-34a nanoparticles

**FIGURE 1:**
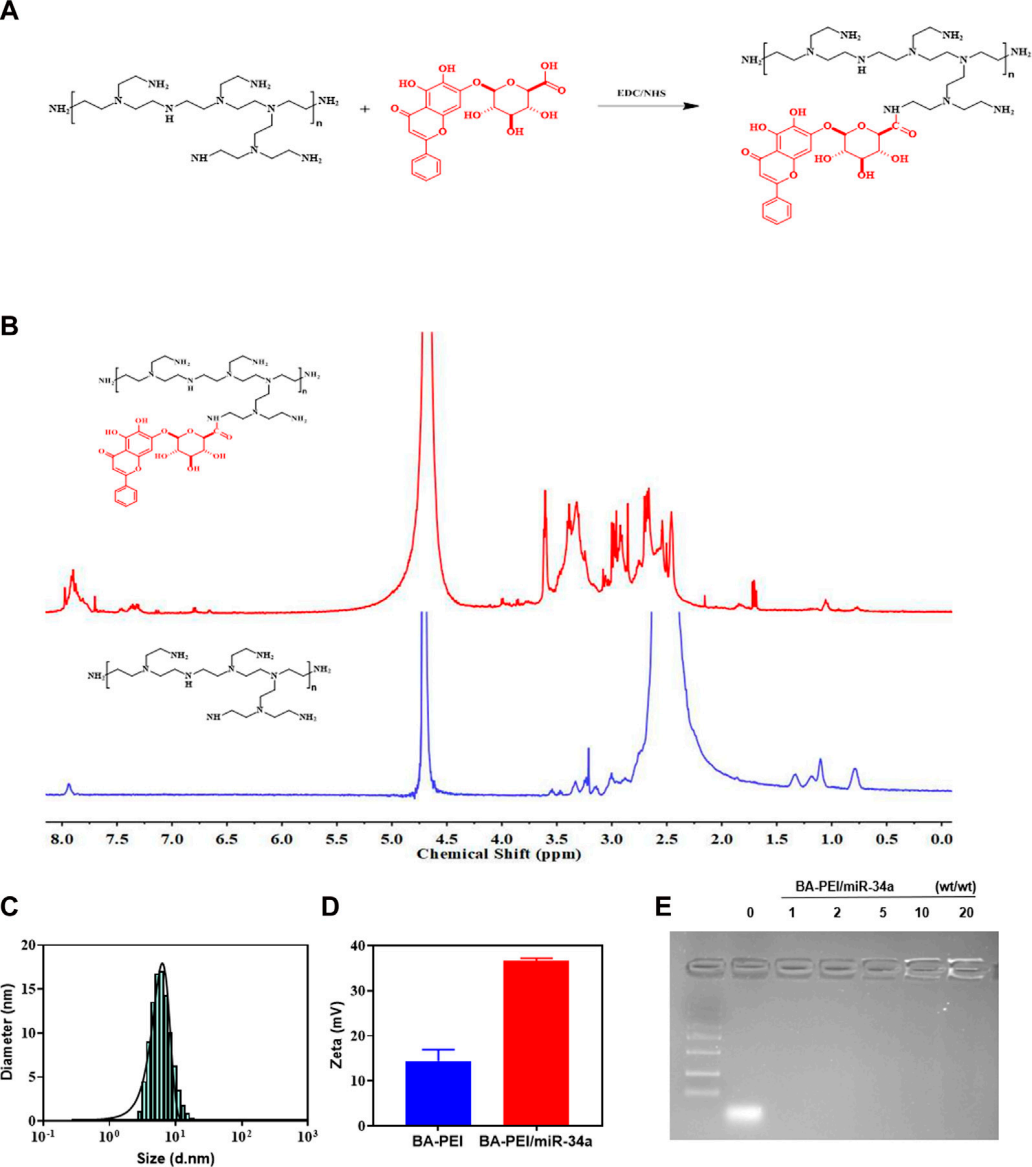
Synthesis of BA-PEI/miR-34a nanoparticles. **(A)** Synthetic route of BA-PEI. **(B)** ^1^H NMR spectra of BA-PEI and PEI. **(C)** Hydrodynamic diameter of BA-PEI/miR-34a complexes. **(D)** Particle size and zeta potential of BA-PEI/miR-34a complexes. **(E)** Gel electrophoresis for BA-PEI/miR-34a at mass ratios of 0, 1, 2, 5, 10, and 20.

BA-PEI was synthesized via a condensation reaction. For the sake of cutting down the PEI25K’s cytotoxicity, we introduced the hydrophobic compound baicalin, which further enhances the cellular ingestion of PEI/miR-34a. In ^1^H NMR, shown in Figure 1B, we can observe the generation of new peaks, indicating that BA has been successfully grafted onto PEI. Under the TEM, we distinctly caught sight of the BA-PEI/miR-34a genetic drug, which had a spherical morphology (Supplementary Figure S1) with a properly smaller particle size compared to BA-PEI measured by dynamic light scattering (DLS; Figure 1C). Furthermore, BA-PEI/miR-34a complexes were formed by electrostatic and hydrophobic interactions, which were characterized through an agarose gel retardation assay. This showed that BA-PEI and miR-34a could form consistently at a mass ratio of 5 (Figure 1E).

### 3.2 *In vitro* cytotoxicity of BA-PEI

**FIGURE 2:**
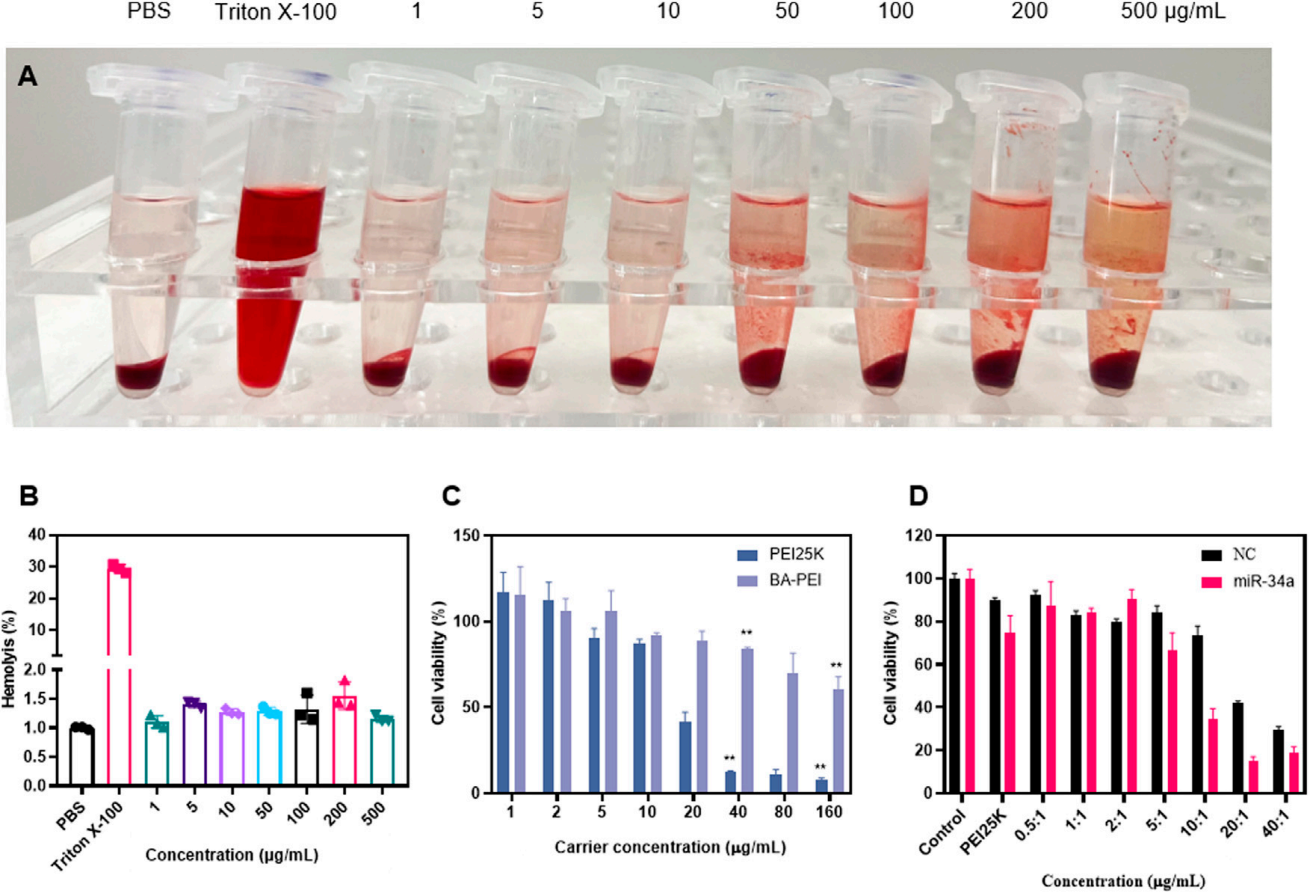
*In vitro* cytotoxicity of BA-PEI and BA-PEI/miR-34a. **(A)** Visual hemolysis assay and **(B)** quantitative hemolysis analysis for several BA-PEI concentrations and Triton X-100-treated mouse erythrocytes. **(C)** Cell viability (%) was conducted by CCK-8 assay for treated A549 cells by different BA-PEI concentrations and PEI. **(D)** Cell viability (%) was conducted by CCK-8 assay for treated A549 cells; the concentration of PEI is approximately 1.33 μg/mL. The concentrations are consistent with the mass ratio of carrier/miR-34a treated in A549 cells; *p <0.05; **p <0.01; ns, not significant.

In order to verify the safety of the material *in vivo*, a hemolysis experiment was used in the biological body, and the results showed that there was no hemolysis phenomenon and it could be safely injected into the tail vein (Figures 2A,B). In CCK-8, when BA-PEI ≤40 μg/mL, there was negligible cytotoxicity to A549 cells (Figure 2C). Compared with PEI25K, the carrier BA-PEI survival rate was higher with good biocompatibility. In order to better observe the transfection efficiency, miR-34a labeled with 5-carboxyfluorescein (FAM) was used for transfection with PEI25K as the control. We found that when the mass ratio is 10, the highest transfection efficiency of BA-PEI is achieved (Figure 2D and Supplementary Figure S2). In addition, in the experiment of living and dead cells, the activity of lipase in cells was detected by calcein-AM, and the living cells were stained with green fluorescence. PI (propyl iodide) detects the integrity of cell membranes and stains dead cells, showing red fluorescence. As shown in Supplementary Figure S3, the staining results showed that compared with PEI25K/miR-34a and the empty carrier BA-PEI, more dead cells were observed in the BA-PEI/miR-34a complex.

### 3.3 Anti-proliferation effect of BA-PEI/miR-34a nanoparticles

**FIGURE 3:**
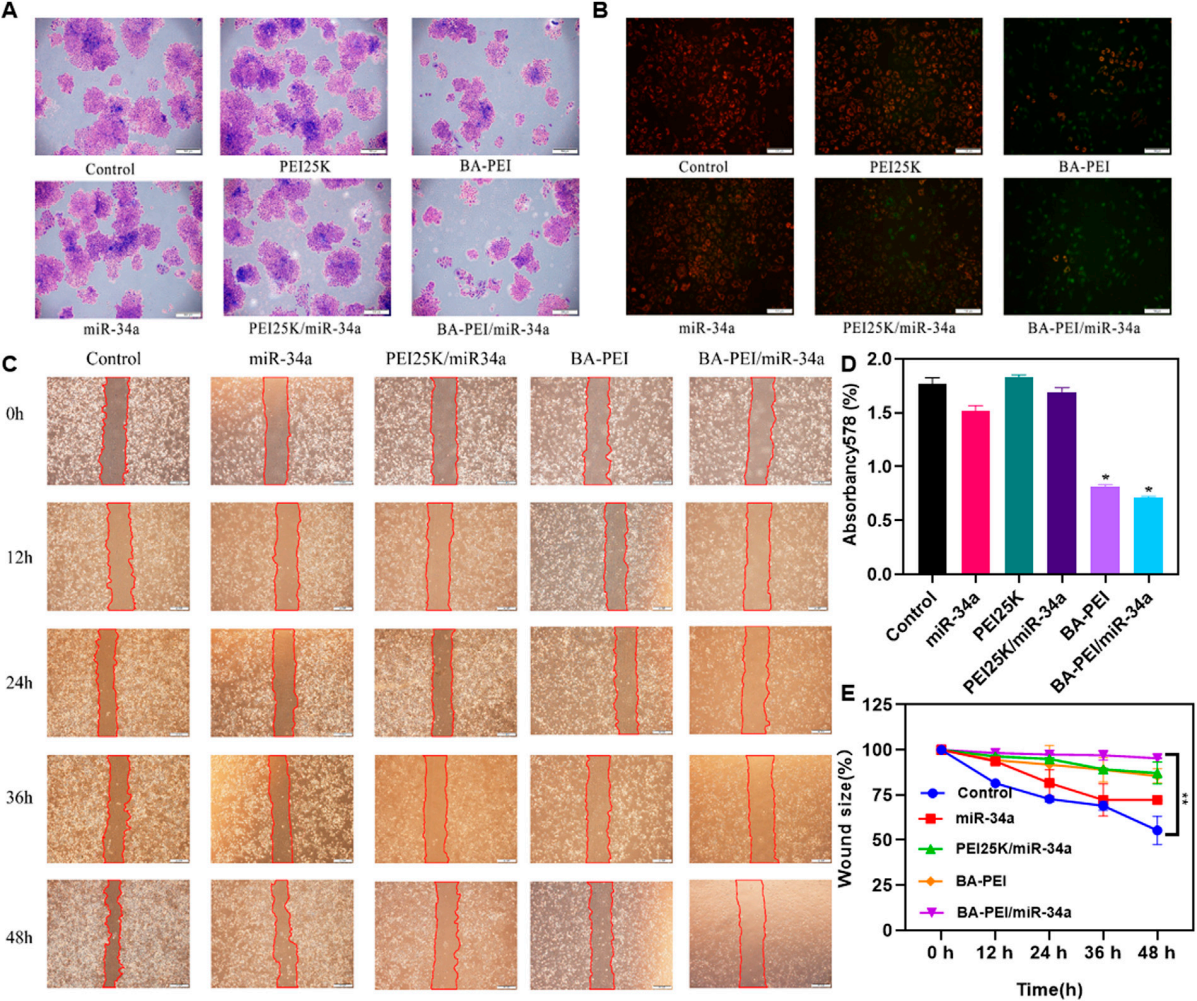
Anti-proliferation effect of BA-PEI/miR-34a nanoparticles. **(A)** One week of cell cloning results of A549 pre-treated with PEI, BA-PEI, miR-34a, PEI/miR-34a, and BA-PEI/miR-34a for 48 h. **(B)** JC-1 of A549 cells after treating with PEI, BA-PEI, miR-34a, PEI/miR-34a, and BA-PEI/miR-34a for 48 h. **(C)** Wound healing assay and **(D)** Quantitative analysis of A. **(E)** quantitative wound size in A549 cells transfected by carrier/miR-34a complexes at 0, 12, 24, 36, and 48 h. The data are shown as mean values ±SEM. *p <0.05; **p <0.01; ns, not significant. Scale bar = 200 μm.

In order to clarify the anti-proliferation effect of BA-PEI/miR-34a nanoparticles, we conducted a cell colony experiment, as shown in Figures 3A,D. Compared with PEI25K/miR-34a and the empty carrier BA-PEI, the BA-PEI/miR-34a complex significantly inhibited the formation of cell colonies. In conclusion, due to the inhibitory effect of miR-34a on tumor cells, BA-PEI-mediated miR-34a transfection has a more effective inhibitory effect on the proliferation of tumor cells. The membrane-permeable JC-1 dye is crucial to monitoring mitochondrial health. In comparison with the control or BA-PEI-treated cells, BA-PEI/miR-34a can effectively achieve anti-tumor effects by inducing mitochondrial damage (Figure 3B). Meanwhile, we used a scratch test to observe the growth of tumor cells after BA-PEI/miR-34a intervention, and we found that the fusion speed of cells in the group of BA-PEI/miR-34a was significantly slower than that in the normal group, showing that BA-PEI/miR-34a could restrain A549 migration (Figures 3C,E).

### 3.4 BA-PEI/miR-34a induced apoptosis of tumor cell A549

**FIGURE 4:**
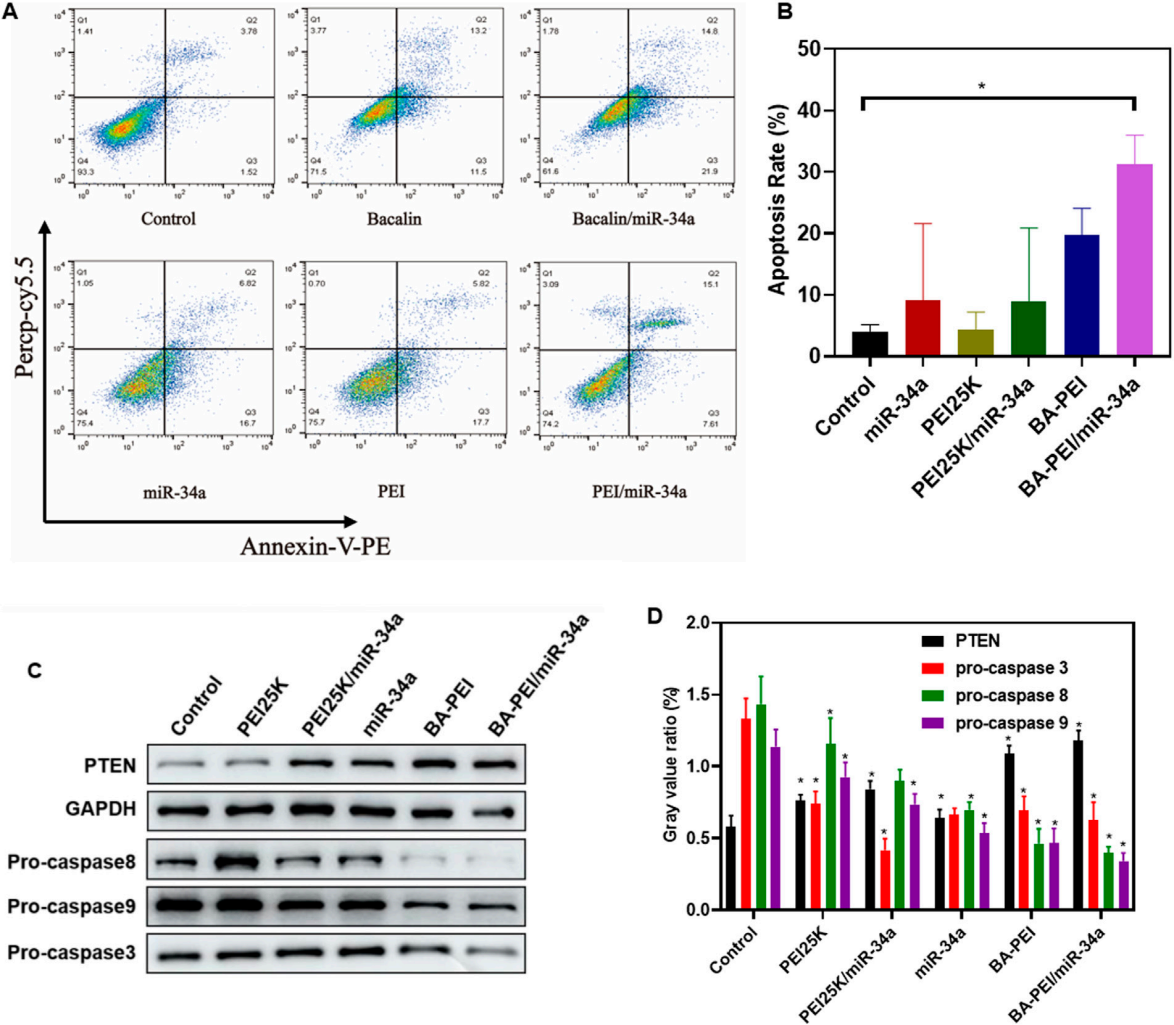
BA-PEI/miR-34a induced apoptosis of tumor cell A549. **(A)** Cell apoptosis analysis using flow cytometry, based on the control, miR-34a, PEI, BAPEI, PEI/miR-34a, and BA-PEI/miR-34a. **(B)** Quantitative analysis of **(A)**. **(C)** Western blots used to analyze the apoptotic protein (pro-caspase-3, -8, and -9 and PTEN), based on the control, miR-34a, PEI, BA-PEI, PEI/miR-34a, and BA-PEI/miR-34a. **(D)** Quantitative analysis of **(C)**. *p <0.05; **p <0.01; ns, not significant.

In order to further investigate the tumor-killing effect of BA-PEI/miR-34a, we conducted the following research. Annexin V-FITC/PI staining, evaluated by flow cytometry, detected the apoptosis rate induced by BA-PEI/miR-34a. As shown in Figure 4A, the apoptosis rates of the empty vector BA-PEI and PEI25K/miR-34a were close to, but lower than that of BA-PEI/miR-34a, manifesting that both miR-34a and baicalin could effectively promote apoptosis and that baicalin and miR-34a acted on tumor cells together, showing the greatest advantage of their synergistic effect. Therefore, we detected the expression variation of proteins to further elucidate the apoptosis-induced pathway by BA-PEI/miR-34a. BA-PEI/miR-34a can significantly reduce the expression of pro-caspase-3 and accelerate the transformation of pro-caspase-3 to caspase-3, which has been considered to be a crucial medium in the caspase family-related apoptotic cascade (Yang et al., 2012). In addition, the alteration of pro-caspase-8 and pro-caspase-9 were decreased after BA-PEI/miR-34a transfection, indicating that caspase-8 and caspase-9 were activated by the cleavage of their precursors. Fundamentally, it has been established that caspase-8 is activated based on death receptor-dependent pathways and caspase-9 through mitochondrial pathways (Wang et al., 2018; Dong et al., 2019). Therefore, we conclude that BA-PEI/miR-34a may cure cancer through death receptor-dependent pathways and mitochondrial pathways and PTEN signaling pathways, which are well-studied tumor inhibition pathways (Jiang and Liu, 2009). PTEN signal transduction inhibits the origin and development of tumors through a variety of mechanisms, including promoting the death of cancer cells and inhibiting cancer cell propagation, migration, and invasion. The consequence was determined by Western blotting. In comparison with the control group, the cells treated with BA-PEI/miR-34a significantly improved the expression of PTEN, indicating that the upregulation of PTEN could inhibit the transfer of cancer cells. It was further confirmed that BA-PEI/miR-34a could regulate tumor suppressor genes to induce cell apoptosis.

### 3.5 *In vivo* anti-tumor efficacy of BA-PEI/miR-34a nanoparticles

**FIGURE 5:**
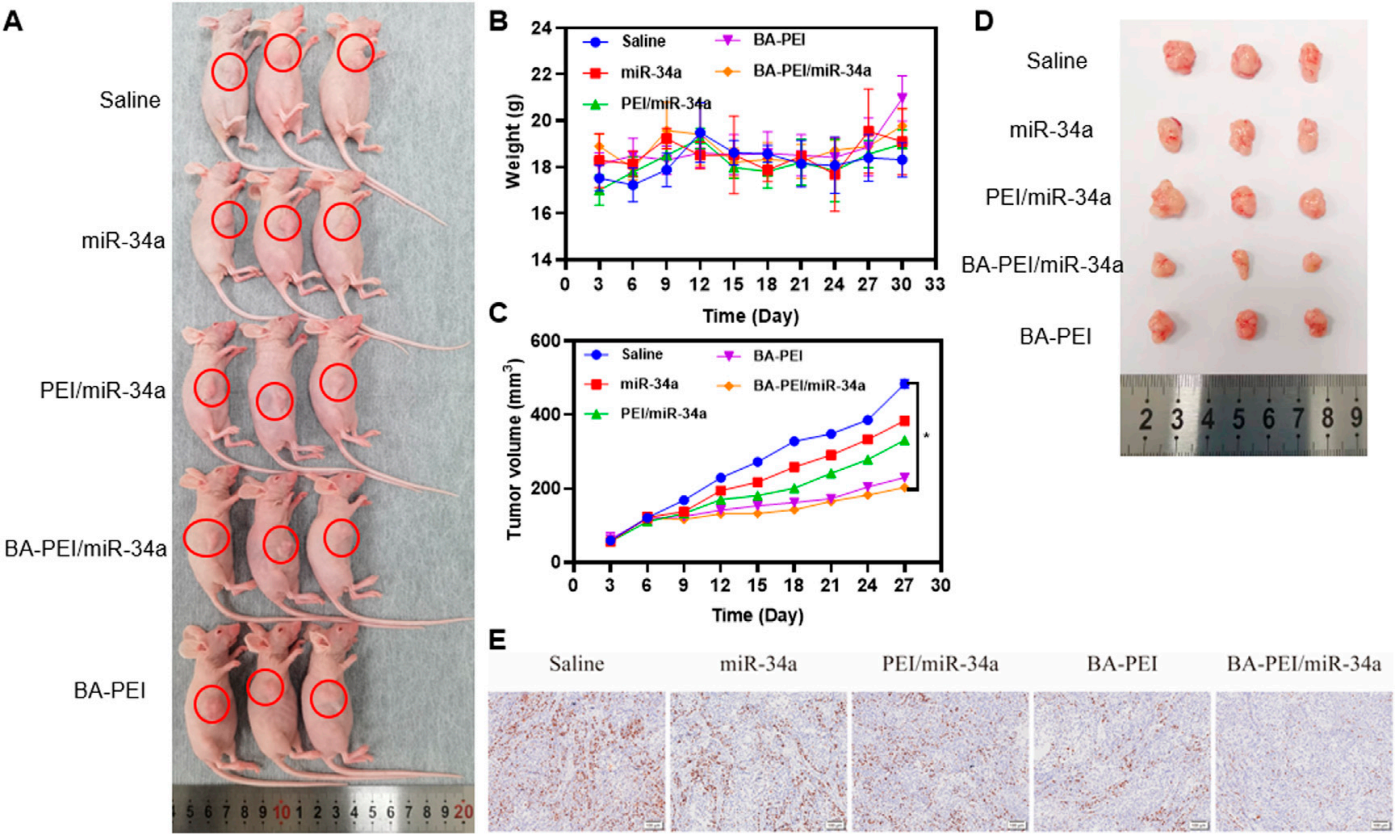
*In vivo* cytotoxicity of BA-PEI/miR-34a. **(A)** Subcutaneous A549 tumor-bearing mice model intravenously administrated with nanoparticles containing miR-34a. **(B)** Body weight of mice during the therapy. **(C), (D)** Tumor volume profiles administrated with saline miR-34a, PEI/miR-34a, BA-PEI/miR-34a, and BA-PEI, with a dose of 0.5 mg/kg every 3 days (*n* = 5). **(E)** Characteristic immunohistochemistry staining of Ki-67 of tumor organs from different complexes treating nude mice after their euthanization. *p <0.05; **p <0.01; ns, not significant. The scale bar is 100 μm.

In order to assess the inhibitory efficacy of BA-PEI/miR-34a complexes, we intravenously administered A549 cells in subcutaneous xenograft nude mice. The established subcutaneous xenograft model is demonstrated in Figure 5A. Moreover, the control group showed rapid tumor growth. When the average tumor volume in the control treated group reached 500 mm^3^, all the mice were euthanized and photographed. As shown in Figures 5C,D, compared with the miR-34a group, mice treated with PEI/miR-34a and BA-PEI/miR-34a exhibited reduced tumor volumes, due to the accumulation of BA-PEI/miR-34a in the cancer regions. Additionally, to further assess the bio-toxicity of the complexes, the organs were harvested and stained with H&E after the treated mice were euthanized. The kidneys, lungs, spleens, hearts, and livers were stained with H&E. Also, there were no evident pathological alterations in any of the treated groups (Supplementary 2). Furthermore, we measured the body weight of the treated mice every 3 days, but no notable significance was observed in the different tumor-bearing mice (Figure 5B) because no system damage occurred after the intravenous injection of the carriers. These results certified that carrier-mediated transfection had relatively low bio-toxicity for the organs of nude mice, representing an attractive utilization of BA-PEI for clinical use. Furthermore, we performed Ki-67 IHC staining on the tumor sections to illustrate the anti-tumor response of different nanoparticles containing miR-34a. The naked miR-34a group showed an intact structure and dense cell arrangement, and the high positive and positive staining of Ki-67 in the tumor nuclei was observed (Figure 5D), indicating rapid tumor growth *in vivo*.

## 4 Conclusion

In conclusion, baicalin-modified PEI25K successfully constructed a traditional Chinese medicine composite BA-PEI vector to deliver miR-34a and achieve a synergic anti-tumor effect. Compared to PEI25K, BA-PEI showed a relatively high capacity for taking miR-34a into the cells and a low toxicity to them. Due to the triumphant expression of miR-34a in cells and animals and the cooperation with baicalin, BA-PEI/miR-34a significantly restrains their proliferation and migration, achieving the purpose of cancer therapy by inducing apoptosis and stalling the cell cycle in the S phase. Therefore, baicalin-modified PEI25K may be an efficient gene carrier for the therapy of cancer and other malignant diseases.

## Data Availability

The original contributions presented in the study are included in the article/Supplementary Material; further inquiries can be directed to the corresponding authors.
