# Correction: Baicalin-modifed polyethylenimine for miR-34a efficient and safe delivery

**DOI:** 10.3389/fbioe.2025.1631667

**Published:** 2025-07-15

**Authors:** Yingying Wang, Baiyan Wang, Yangfan Xiao, Qingchun Cai, Junyue Xing, Hao Tang, Ruiqin Li, Hongtao Zhang

**Affiliations:** ^1^ Medical College, Henan University of Chinese Medicine, Zhengzhou, Henan, China; ^2^ National Health Commission Key Laboratory of Cardiovascular Regenerative Medicine, Heart Center of Henan Provincial People’s Hospital, Fuwai Central China Cardiovascular Hospital and Central China Branch of National Center for Cardiovascular Diseases, Central China Fuwai Hospital of Zhengzhou University, Zhengzhou, Henan, China; ^3^ Department of Clinical Lab, The Third Affiliated Hospital of Henan University of Chinese Medicine, Zhengzhou, China; ^4^ Academy of Chinese Medicine, Henan University of Chinese Medicine, Zhengzhou, Henan, China; ^5^ Blood Purification Center, The People’s Hospital of Zhengzhou University, Zhengzhou, China; ^6^ Blood Purification Center, Henan Provincial People’s Hospital, Zhengzhou, China; ^7^ Institute of Nephrology, Mathura, Henan, China; ^8^ Department of Nephrology Henan Provincial People’s Hospital, Zhengzhou, China

**Keywords:** baicalin, lung cancer, gene therapy, miR-34a, hydrophobic modification

In the published article, there was an error in Western blot of Figure 4C as published. An extra GAPDH band image was included in the original Figure 4C. The corrected Western blot of Figure 4C and its caption FIGURE 4 BA-PEI/miR-34a induced apoptosis of tumor cell A549. (C) Western blots used to analyze the apoptotic protein (pro-caspase-3, -8, and -9 and PTEN), based on the control, miR-34a, PEI, BA-PEI, PEI/miR-34a, and BA-PEI/miR-34a. appear below.

**FIGURE 4:**
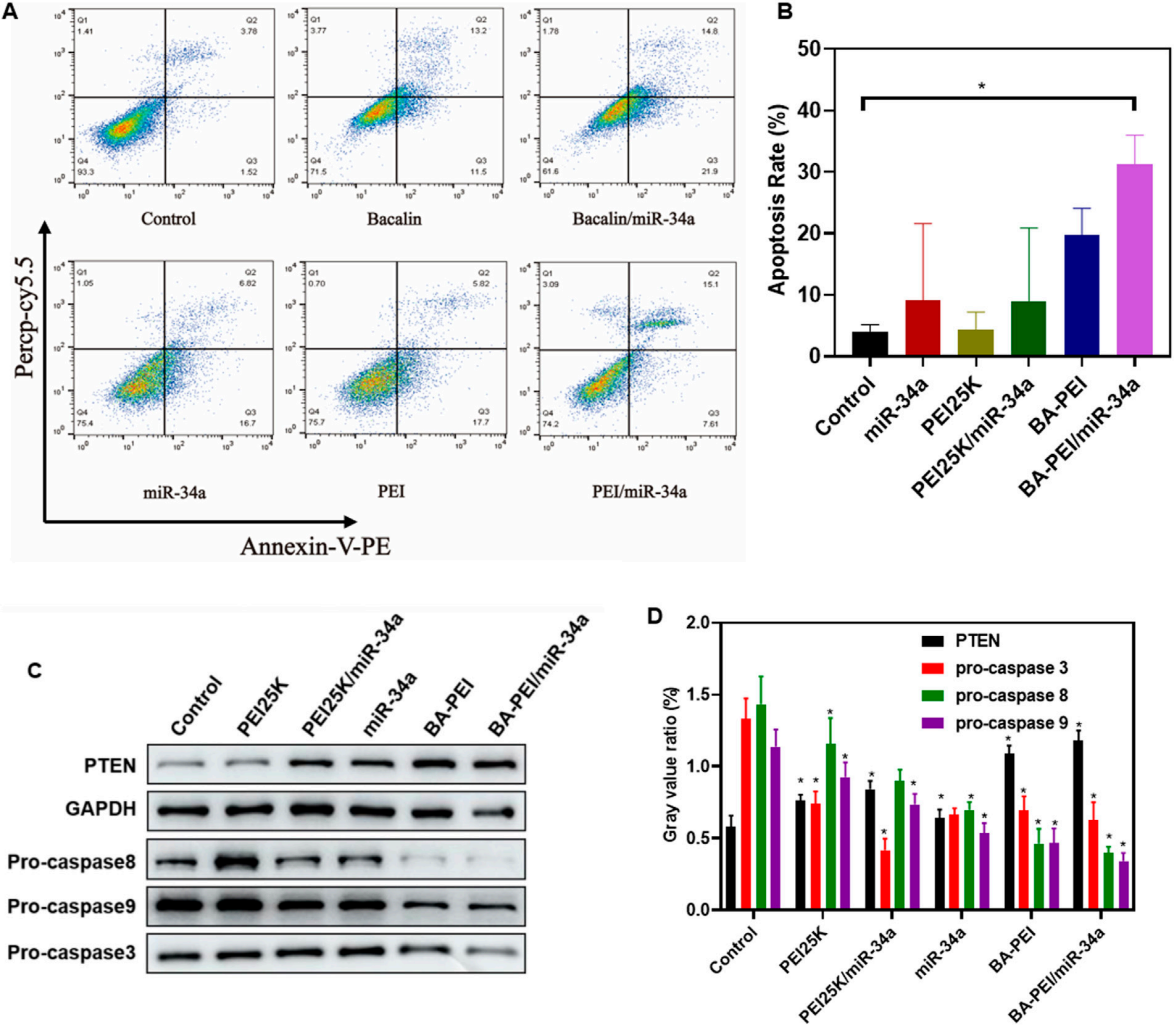
BA-PEI/miR-34a induced apoptosis of tumor cell A549. **(A)** Cell apoptosis analysis using flow cytometry, based on the control, miR-34a, PEI, BAPEI, PEI/miR-34a, and BA-PEI/miR-34a. **(B)** Quantitative analysis of **(A)**. **(C)** Western blots used to analyze the apoptotic protein (pro-caspase-3, -8, and -9 and PTEN), based on the control, miR-34a, PEI, BA-PEI, PEI/miR-34a, and BA-PEI/miR-34a. **(D)** Quantitative analysis of **(C)**. *p <0.05; **p <0.01; ns, not significant.

The original version of this article has been updated.

