# Supplementary material for: Baicalin-modified polyethylenimine for miR-34a efficient and safe delivery
Source: Front Bioeng Biotechnol. 2023 Nov 3;11:1290413. doi: 10.3389/fbioe.2023.1290413 (PMC10656686; doi:10.3389/fbioe.2023.1290413)
Supplement: Supplementary file 1 [file DataSheet1.docx]

**Materials and Methods**

**Materials**

RPMI1640 (Solarbio, Beijing), Fetal Bovine Serum (FBS) (BI, Israel). CCK-8 (AbMole, Hangzhou, China). 1% Crystal Violet was purchased from Solarbio (Beijing, China). Monoclonal antibodies against procaspase-3, procaspase-8, procaspase-9, PTEN, GAPDH, Horseradish peroxidase (HRP) -labeled goat anti-rabbit or anti-mouse IgG purchased from Abcam (Shanghai, China). The PVDF membrane with a pore size of 0.22 μm was purchased from Millipore Co., Bedford, USA. Annexin V-FITC/PI Apoptosis detection kit was provided by thermo (Shanghai, China). Cell Cycle kit from BestBio (Shanghai, China). LIVE/DEAD kits for mammalian cells were purchased from Beyotime (Shanghai, China). Mitochondrial membrane potential detection kit (JC-1) was purchased from Beyotime (Shanghai, China). The miR-34a and FAM labeled miR-34a were obtained from GenePharma Co. (Suzhou, China).

miR-34a Forward: 5'-UGGCAGUGUCUUAGCUGGUUGU-3';

Reverse: 5' -AACCAGCUAAGACACUGCCAUU-3 ';

NC Forward: 5'-UUCUCCGAAAACGUGUCACGUTT-3';

Reverse: 5'-ACGUGACACGUUCGGAGAATT-3'.

**BA-PEI preparation**

Dissolve Baicalin in MES, add 1M of NaOH until the powder is completely dissolved, Baicalin with EDC and NHS was added at 1.2:1:1 molar ratio and stirring at room temperature for 15-30 min. Then PEI was added and stirring for 30min, then the solution was dialyzed in dd H_2_O with a permeable bag (Mw =3500-12000). Finally, the final product BA-PEI was freeze-dried overnight.

**Cube Mito-Ru MOF synthesis**

Lecithin (10 mg), DSPE-PEG-NH_2_ (1.0 mg), and cholesterol (2.0 mg) were dissolved in 3.0 mL of a 1:1 (v/v) chloroform/methanol solvent mixture and the latter was removed by vacuum. Then 2 mg Ru MOF solution (2 mL) was added to the residue and subjected to 300 W ultrasonic treatment for 5 min to obtain the Ru MOF@Liposome. Then 10 mM carbodiimide-HCl (EDC) and 25 mM *N*-hydroxysuccinimide (NHS) were added to 10 mM TPP (pH 7.2) solution. The mixture was incubated at 30 °C with shaking for 15 min and added to the Ru MOF@Liposome solution to obtain the Mito-Ru MOF.

**Animals**

Balb/c nude mice aged 6–8 weeks and each weighing 18–21 g were provided by the Animal Laboratories Center of Zhengzhou University, Zhengzhou, Henan, PRC, and housed at the central facility of the Zhengzhou University School of Medicine under a standard 12 h light/12 h dark cycle. They had *ad libitum* food and water access. The study protocol was approved by the Institutional Ethics Review Committee of Zhengzhou University (No.2021062). The number of animals used and animal suffering were minimized to the greatest extent possible.

**Cell Culture**

Human A549 cells were obtained from the Cell Type Culture Collection of the Chinese Academy of Sciences (Shanghai, China) and cultured in RPMI-1640 medium (BI, Israel) supplemented with 10% fetal bovine serum, 100 U/mL penicillin, and 100 μg/mL streptomycin (Hyclone, Logan, UT, USA). All cells were maintained in a humidified incubator at 37°C and 5% CO_2_.

**Anti-tumor effect of nanoparticles transfection**

The anti-proliferative effect was assessed through CCK-8. A549 cells were planted and incubated in 96 (5×10^3^ cells/well) overnight, then transfected by different nanoparticles in 1640 medium for 6 hours. And the medium was replaced by 10% FBS-containing 1640 for another 48 h. For the CCK-8 assay, 10 μL CCK-8 solution was added to co-culture for 2 h. The absorbance at 450 nm was measured on a Cytation 5 enzyme label (Bio Tek, USA). Cell viability (%) was measured concerning the control based on the formula:

**Live/Dead cell staining**

The cell culture and nanoparticles transfection were implemented as described before. The collected cells were washed with PBS solution, managed with Live/Dead reagents (Calcein/PI) at 37 °C for 30 min, and observed on a fluorescence microscope, IX73 (OLYMPUS, Japan).

**Mitochondrial membrane potential (ΔΨm) assay**

The A549 cells were seeded as described previously. Briefly, cells indicated treatments were incubated with f JC-1 staining solution at 37 °C for 20 min and rinsed twice with PBS. The mitochondrial membrane potential was monitored by determining the relative amounts of dual emissions from mitochondrial JC-1 monomers or aggregates using an Olympus fuorescence microscope. Mitochondrial depolarization is indicated by an increase in the green/red fuorescence intensity ratio

**Inhibitory effect of nanoparticles *in vivo***

The tumor volume and body weight of nude mice were measured every three days, and all mice were sacrificed at 33 days. Then we harvested tumor tissues and organs (including heart, lung, spleen, liver, and kidney) and fixed in 4% (w/v) paraformaldehyde solution. These fixed tissues were placed in paraffin blocks and sliced for hematoxylin and eosin (H&E) staining and immunohistochemistry (IHC) of Ki67. These sections were monitored and captured under a microscope, BX53 (OLYMPUS, Japan). Relative quantitative analysis of high positive, positive, and low positive staining of Ki67 were evaluated via Image J software.

**Statistical analysis**

Data were presented as means ± standard deviation (SD). Statistically significant differences between group pairs were determined with Student’s *t*-test. Two-way analysis of variance (ANOVA) followed by the Bonferroni test detected statistically significant differences among multiple groups. Differences were considered significant at *P <* 0.05. GraphPad Prism v. 7.0 (GraphPad Software Inc., La Jolla, CA, USA) was used for all data processing.


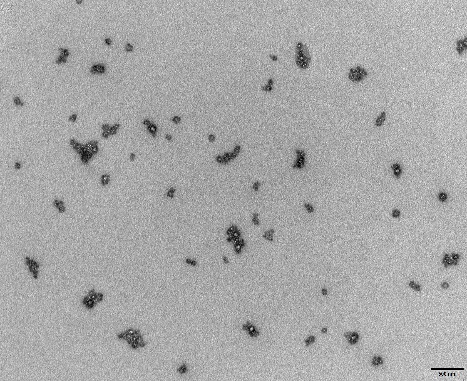


**Figure S1.** morphology of BA-PEI/miR-34a particles under TEM. The scale bar represents 500 nm.


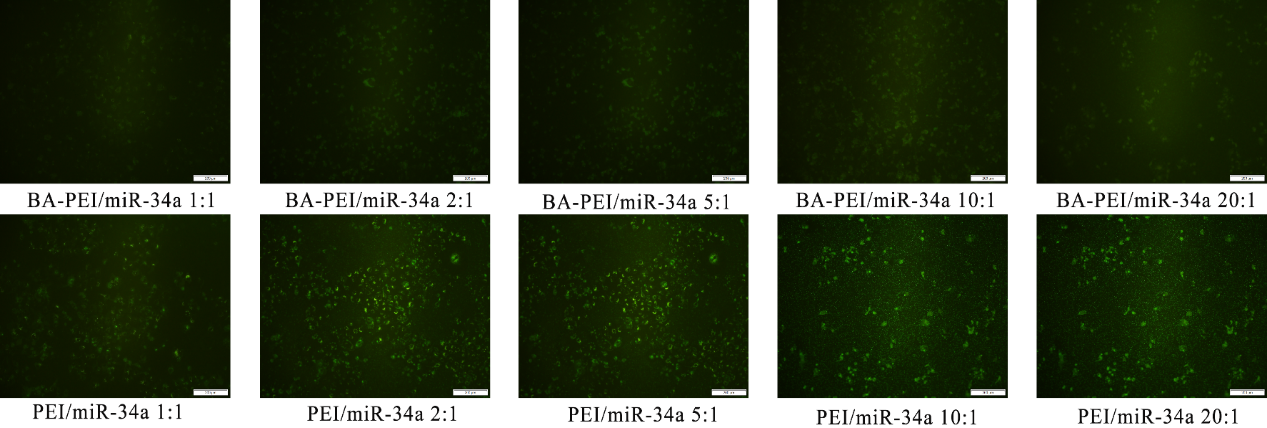


**Figure S2.** The transfection of BA-PEI/miR-34a and PEI/miR-34a particles under fluorescence microscope. The concentrations are consistent with mass ratio of carrier/miR34a treated in A549 cells

**
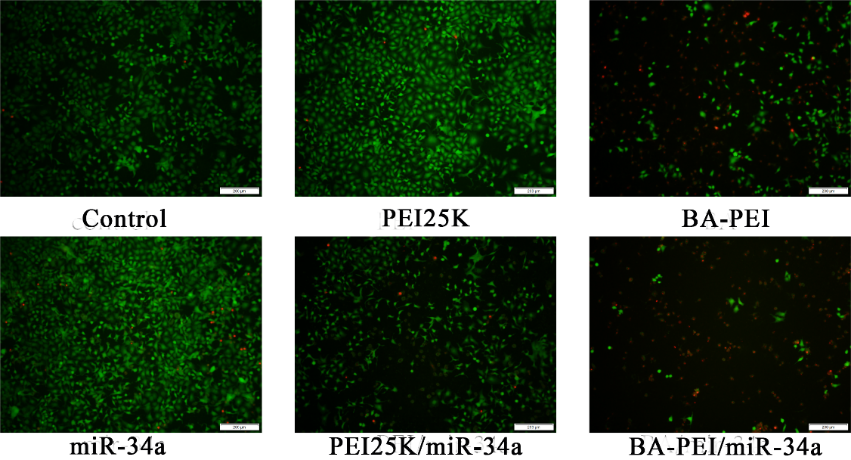
**

**Figure S3.** LIVE–DEAD cell-staining of A549 cell after treating with PEI, PEI/miR-34a, miR-34a, BA-PEI and BA-PEI/miR-34a.

**
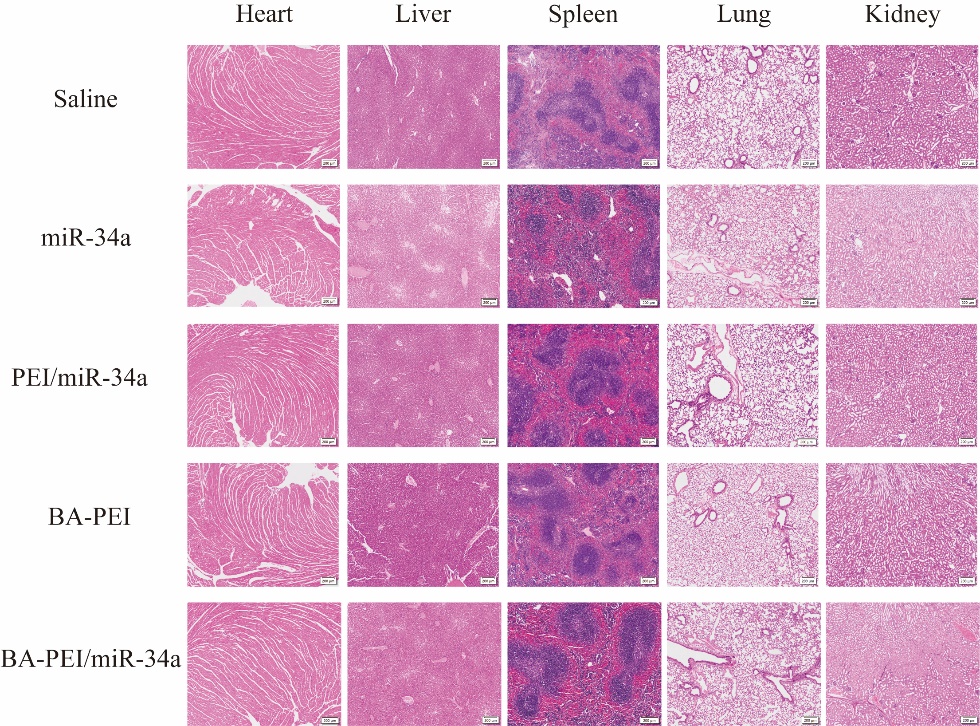
**

**Figure S4.** In vivo toxicity by H&E staining of different organs after treated with several nanoparticles. The scale bar is 100 μm.
